# Cherenkov emissions for studying tumor changes during radiation therapy: An exploratory study in domesticated dogs with naturally-occurring cancer

**DOI:** 10.1371/journal.pone.0238106

**Published:** 2020-08-26

**Authors:** Ashlyn G. Rickard, Hiroto Yoshikawa, Gregory M. Palmer, Harrison Q. Liu, Mark W. Dewhirst, Michael W. Nolan, Xiaofeng Zhang

**Affiliations:** 1 Department of Radiation Oncology, Program of Medical Physics, Duke University School of Medicine, Durham, NC, United States of America; 2 Department of Clinical Sciences, College of Veterinary Medicine, NC State University, Raleigh, NC, United States of America; 3 Comparative Medicine Institute, North Carolina State University, Raleigh, NC, United States of America; 4 Program of Biology, University of North Carolina at Chapel Hill, Chapel Hill, NC, United States of America; 5 Duke Cancer Institute, Duke University, Durham, NC, United States of America; 6 Artificial Intelligence, Marchex Inc., Seattle, WA, United States of America; Colorado State University, UNITED STATES

## Abstract

**Purpose:**

Real-time monitoring of physiological changes of tumor tissue during radiation therapy (RT) could improve therapeutic efficacy and predict therapeutic outcomes. Cherenkov radiation is a normal byproduct of radiation deposited in tissue. Previous studies in rat tumors have confirmed a correlation between Cherenkov emission spectra and optical measurements of blood-oxygen saturation based on the tissue absorption coefficients. The purpose of this study is to determine if it is feasible to image Cherenkov emissions during radiation therapy in larger human-sized tumors of pet dogs with cancer. We also wished to validate the prior work in rats, to determine if Cherenkov emissions have the potential to act an indicator of blood-oxygen saturation or water-content changes in the tumor tissue–both of which have been correlated with patient prognosis.

**Methods:**

A DoseOptics camera, built to image the low-intensity emission of Cherenkov radiation, was used to measure Cherenkov intensities in a cohort of cancer-bearing pet dogs during clinical irradiation. Tumor type and location varied, as did the radiation fractionation scheme and beam arrangement, each planned according to institutional standard-of-care. Unmodulated radiation was delivered using multiple 6 MV X-ray beams from a clinical linear accelerator. Each dog was treated with a minimum of 16 Gy total, in ≥3 fractions. Each fraction was split into at least three subfractions per gantry angle. During each subfraction, Cherenkov emissions were imaged.

**Results:**

We documented significant intra-subfraction differences between the Cherenkov intensities for normal tissue, whole-tumor tissue, tissue at the edge of the tumor and tissue at the center of the tumor (p<0.05). Additionally, intra-subfraction changes suggest that Cherenkov emissions may have captured fluctuating absorption properties within the tumor.

**Conclusion:**

Here we demonstrate that it is possible to obtain Cherenkov emissions from canine cancers within a fraction of radiotherapy. The entire optical spectrum was obtained which includes the window for imaging changes in water and hemoglobin saturation. This lends credence to the goal of using this method during radiotherapy in human patients and client-owned pets.

## Introduction

Physical interactions between oxygen and radiation are critical to the success of megavoltage X-ray therapy (RT). This is because hypoxic tumor cells are 3X more radioresistant than well-oxygenated tumor cells; thus, tumor hypoxia detrimentally affects the RT patients’ prognosis [1]. With intensity-modulated radiation therapy (IMRT) where radiation can target sub-volumes of tumors (sometimes referred to as “dose-sculpting” or “dose-painting”), honing in on hypoxic regions to deliver a radiation boost could increase local tumor control and long-term survival [2, 3]. For this strategy to work, the hypoxic sub-volume needs to remain in the same spot during therapy delivery. Pre-clinical and emerging clinical data suggest that hypoxic subvolumes may move at a frequency that would preclude hypoxia targeting, but the best proof of feasibility would come if measurements could be made during a fraction of radiotherapy. Thus far, attempts to achieve such measurements has been challenging [4, 5] While chronic hypoxia generally stays in the same area, cycling hypoxia can change both spatially and temporally on the order of hours to days, requiring serial imaging to quantify changes. Most imaging modalities are ill-suited for measuring such changes.

Bussink, *et al*, reported that there is an acute change in perfusion and the hypoxic fraction in a preclinical, squamous-cell carcinoma xenograft model. Immediately after radiation (within 2h), perfusion increases before significantly decreasing to baseline a week later; the hypoxic fraction decreases and then increases [6]. These changes might begin during radiation delivery, and imaging them could be achieved with MRI+LINAC systems. However, these are not widely available, interpreting functional tissue changes is challenging, and imaging would take much longer than a single fraction of RT delivery [7]. Functional MR images generally require multiple pulse sequencing, resulting in images sessions up to 30min; whereas, Cherenkov imaging records all data in real-time. Moreover, in radiobiological experiments performed by Kallman in the mid-1970s, he shows that the kinetics of reoxygenation vary drastically between different tumor types: osteogenic sarcoma, fibrosarcoma, and mammary carcinoma [8]. Due to technological and ethical limitation, these experiments were only performed on mice with murine tumors, but it suggests to truly understand the relationship between hypoxia and radiation, it is necessary to gather data from every type of tumor, for every RT fraction and, especially, for every patient.

Hypoxia is comprised of two components: a chronic element that remains hypoxic for long periods and a cyclical element where hypoxic regions vary periodically on the order of minutes to days [5], leading to temporal and spatial variations in tumor hypoxia. Cycling hypoxia is a challenge to overcome with respect to delivering high doses of radiation to specific, hypoxic areas. High-throughput, noninvasive, and quantitative imaging is critical for accurately determining the spatial and temporal hypoxic fraction. The most common method for directly quantifying hypoxia clinically is using an oxygen-sensitive radionuclide with positron emission tomography (PET). However, this system yields low-resolution, low-contrast images that contribute radiation dose to patients (especially when combined with computed tomography), making multiple imaging sessions impractical and potentially hazardous [9, 10]. Notably, oxygen-sensitive radioisotopes tend to have long clearance times (up to 3h), making them unsuitable for cycling hypoxia measurements as well as high uptake in normoxic tissue [5]. Magnetic resonance imaging (MRI) has several pulse sequences suited for indirectly monitoring oxygen, but the expense and challenge in quantification makes this choice unsuitable for combining with RT [11–13]. Hemoglobin saturation measurements are commonly made in clinical environments using either a pulse oximeter or an arterial-blood gas test. The former method is ill-suited for monitoring broad areas of hemoglobin oxygen content, and would be difficult to place on a tumor during radiation. There are several less-common optical spectroscopic methods for measuring hemoglobin saturation during surgery [14, 15] or when the area of interest is directly visible [16]. These methods are powerful and useful for their specific applications, but would be difficult to utilize for large areas of tumor that are beneath skin or tissue.

Recently, there has been progress in using Cherenkov energy as a method for monitoring biological changes (like oxygen) during RT [17–19]. Cherenkov emissions occur as a natural byproduct of RT. As high-energy, charged particles enter a dielectric medium at a speed higher than the local phase velocity of light, optical photons are emitted. In several *in vivo* models, Cherenkov emissions have been recorded during RT [18]. The emitted photons extend from the ultra-violet into the near-infrared spectrum (favoring the lower with a higher intensity), making their absorption and scattering coefficients low and useful for obtaining information on deeper tissues [17]. Most of the work in mega-voltage RT-produced Cherenkov emissions is in dosimetry measurements where the Cherenkov intensity is linearly proportional to the radiation dose [17, 20]. However, in a preclinical study in rats by Zhang, *et al*, Cherenkov emissions directly correlated with independent measurements of tissue oxygenation [17]. Researchers 1) used the known scattering and spectral properties between Cherenkov emissions and hemoglobin saturation to obtain a calibration curve, 2) irradiated the healthy flank muscle in rats while breathing known oxygen concentrations, 3) recorded Cherenkov intensity, and 4) simultaneously measured arterial oxygen concentrations and local-muscle, oxygenated hemoglobin saturation [17]. These combined measures provide the necessary data to positively correlate tissue oxygenation with Cherenkov intensity. A similar experiment in nude mice and phantoms by Axelsson, *et al*, also describes using Cherenkov emission intensity during RT with spectroscopy to calculate Hb saturation [18].

These studies show that it is possible to reliably determine hemoglobin saturation via Cherenkov emissions during RT in a preclinical setting. However, it is uncertain if a similar system could be integrated into the clinic. Thus, we designed a pilot clinical study to observe Cherenkov emissions in six pet dogs with naturally-occurring shallow, soft-tissue neoplasms that were scheduled to undergo routine external beam irradiation as part of their clinical veterinary care. The goals were to evaluate 1) the integration of this Cherenkov imaging system into the clinical workflow and 2) determine if the Cherenkov emissions change during RT.

## Methods and materials

### Animals

Six client-owned, tumor-bearing dogs were enrolled in a prospective clinical study. Each dog had a naturally-occurring cancer, and was scheduled to undergo routine clinical tumor irradiation (**Table 1**). The tumor types include a mast-cell tumor on the cranial left foreleg, a mast-cell tumor on the right buccal mucosa, a fibromatous epulis in right maxillary arcade, a poorly differentiated spindle cell tumor in left mandible, and two soft tissue sarcomas: one on the right hip and the other on the right cranial shoulder. The study was performed with approval of the NC State Institutional Animal Care and Use Committee; each pet owner also provided written consent for study enrollment.

**Table 1:** Description of canine subjects. Data were acquired from 6 dogs with different cancers and tumor locations; data from 5 of 6 dogs were included in the full analysis.

sCase	Dose per fraction (Gy)	Fractionation	Gantry Angles	Number of Subfractions per Fraction	Number of Completed Fractions	Tumor Location	Tumor Histology	Study Inclusion
#1	6	5, twice weekly	40^∘^ and 290^∘^	6	All	Soft tissue sarcoma	Right cranial shoulder	Included
#2	6	5, weekly	70^∘^ and 250^∘^	6	All	Soft tissue sarcoma	Right hip	Included
#3	8	3, on days 0, 7, and 21	0^∘^	3	All	Right maxillary buccal mucosa	Mast cell tumor	Included
#4	4	4, weekly	45^∘^ and 315^∘^	6	3	Poorly differentiated spindle cell neoplasm	Left mandible	Included in Summary Data
								Tumor difficult to visualize for detailed analysis
#5	8	3, weekly	30^∘^ and 210^∘^	6	2	Caudal right maxillary arcade	Fibromatous epulis	Included in Summary Data
								Tumor difficult to visualize for detailed analysis
#6	8	3, on days 0, 7, and 21	0^∘^ and 180^∘^	6	All	Left lateral elbow	Mast cell tumor	Not Included; incomparable camera settings used

### Radiation therapy planning

All dogs were anesthetized and underwent CT simulation. Pre and post-contrast CT images were imported into a RT planning system (Varian Eclipse version 11.0.31, Varian Medical Systems, Varian Medical Systems, Inc. Palo Alto, CA). Organs at risk, as well as gross tumor volume (GTV) clinical target volume (CTV) and planning target volumes (PTV) were approved by the attending veterinary radiation oncologist. Forward planning was used, with 6 megavoltage (MV) X-ray beams. Plans were made on pre-contrast CT images but post-contrast images were used to aid target delineation. Dose calculation was performed using analytic anisotropic algorithm (AAA) with a 0.25 cm calculation grid size. Primary jaws and multi-leaf collimators were used to shape the fields; intensity-modulation was not used. A 1.0cm water-equivalent bolus transparent to Cherenkov emissions (Polygel LLC, Whippany, NJ) and physical wedges were used if dosimetrically beneficial. In most of the cases, 2–3 parallel/orthogonal, co-planar 6MV beams were used. The planning goal was to cover 95% of PTV with the prescribed dose while avoiding excessive hotspot in the organs at risk (ie. Skin) based on an institutional normal tissue tolerance. Dose normalization was used if required to achieve the goal. Second check of monitor unit calculations was performed using commercial software (Rad Calc, LifeLine Software, Inc. Austin, TX), and plans were reviewed by a staff medical physicist. Next, each dose fraction was divided into 3–4 equally-sized sub-fractions. For example, if a plan was scheduled to deliver 100 monitor units from each of 3 beams at different gantry positions, 4 subfractions would have been generated. Each subfraction would deliver 25 monitor units from each gantry position. For treatment delivery, each subfraction was given at the usual dose rate (600 monitor units per minute). Subfractions were temporally separated by 5 minutes. This approach of splitting the individual fractions allowed us to evaluate intrafraction changes in tumor oxygen status and to provide time to change beam wedges or check on the dog.

RT was delivered to the anesthetized patient using a linear accelerator (Varian Novalis TX^TM^, Varian Medical Systems). Prior to treatment, the fur was clipped on and around the tumor if necessary. Analgesics such as non-steroidal anti-inflammatory drugs and opioids were administered to minimize patient discomfort. After each treatment, patients were sent home with analgesics if deemed beneficial by attending veterinarians.

### Cherenkov imaging setup and acquisition

The Cherenkov-imaging system includes a laptop for image acquisition (ASUS) with the C-Dose software (C-Dose Research 2.03) installed. This laptop also has the software for Oxylite data acquisition (WINDAQ, DATAQ Instruments, v. 2.99). The DoseOptics Camera (C-Dose Research) was equipped with a lens (Nikon AF NIKKOR 50mm 1:1.8 D), placed on a tripod, and connected to the control box from which the radiation trigger and fiber optics cables were connected. Using an optical repeater (FireNEX-5000H), the fiber optics cable connected to the computer via USB. The Oxylite (Oxford Optronix) had two pre-calibrated probes for each animal (tumor and normal tissue), and it connected to the laptop via a long USB cable for recording the data. All the cables fit underneath the LINAC shielded door to the laptop.

**Fig 1:**
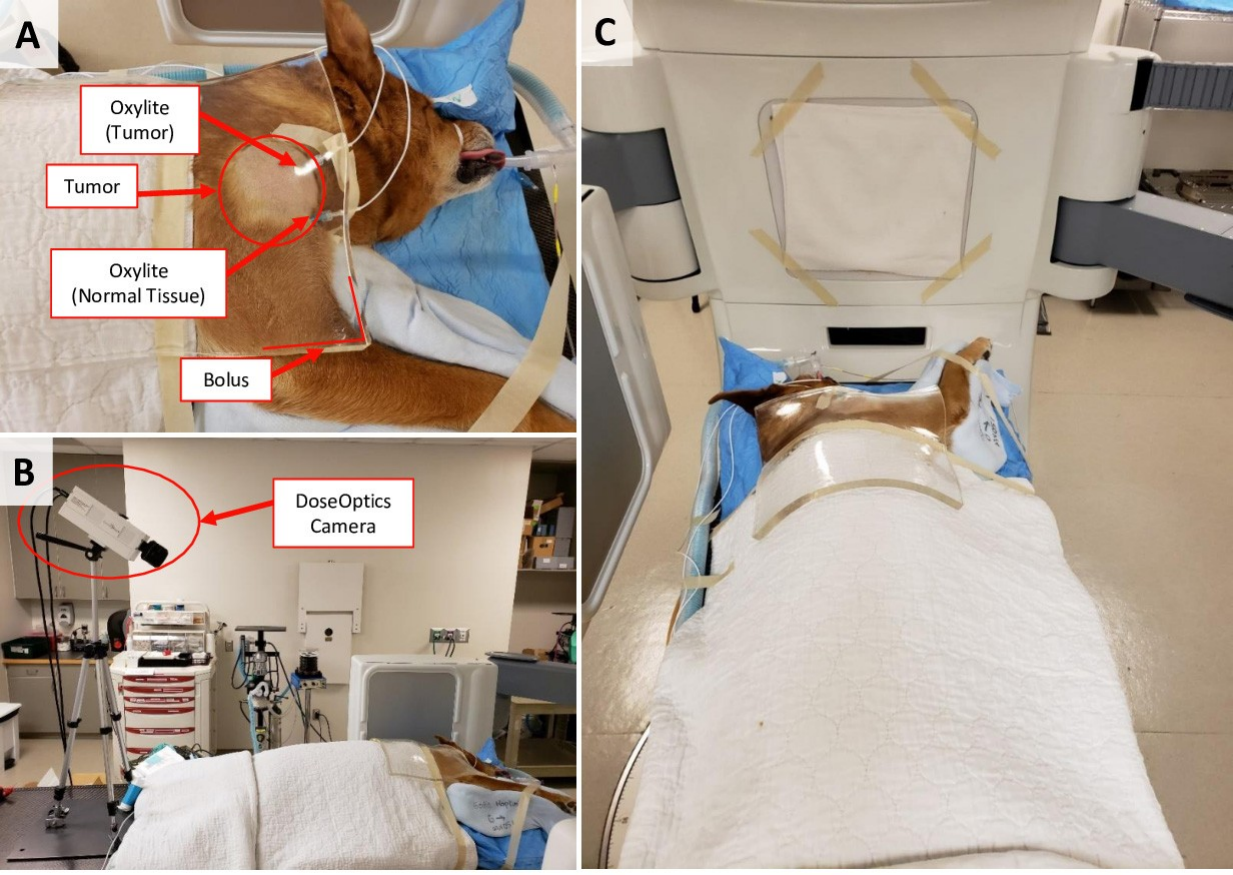
Setup of canine and camera for multibeam radiotherapy. **A)** A close-up image of the tumor on the shoulder of this dog demonstrates where the Oxylite probes were placed within the tumor and normal tissue. On top of the tumor is a Cherenkov-transparent bolus. **B)** The camera was placed on the table behind the subject so the gantry would not interfere with the camera, and any table movements would maintain the same camera-tumor position. **C)** From the camera’s point-of-view, the tumor is visible.

Using the DoseOptics camera setup, we imaged canine subjects during radiation delivery. For larger tumors that were not located in the mouth, setup was straightforward. Generally, the camera was placed on the bed, behind the animal. This allowed the gantry to move unhindered for a multi-beam radiation plan, and this setup retained the tumor-camera position should the table need to be adjusted after imaging (**Fig 1**). While veterinary providers anesthetized the animal, the camera was set up and focused. Then, after KV or cone-beam CT imaging to confirm that the animal was in the proper position, the table was rotated or adjusted without spoiling the camera focus. In some dogs, the tumor location necessitated placement of the camera lateral to the table. This changed the workflow for setup verification subtly; the animal was anesthetized, imaged and the table adjusted, then the camera was focused. Due to the large field-of-view of the DoseOptics camera and its sensitivity to Cherenkov emissions, camera could be placed at a distance to allow unimpeded gantry movement. Some gantry angles might have obstructed the camera’s view, but this issue never arose during this pilot study due to the flexibility in camera positioning.

Because we are interested in how changes in pO_2_ during RT are reflected in Cherenkov emissions, we measured tumor and normal tissue pO_2_ via Oxylite probes for comparison. Two Oxylite probes were placed in each dog: one in the tumor and the other in surrounding normal tissue–both probes were positioned to receive full radiation dose–i.e., within the planning target volume. Proper probe placement was confirmed via visual inspection of a pre-treatment cone-beam CT by the attending radiation oncologist.

**Fig 2:**
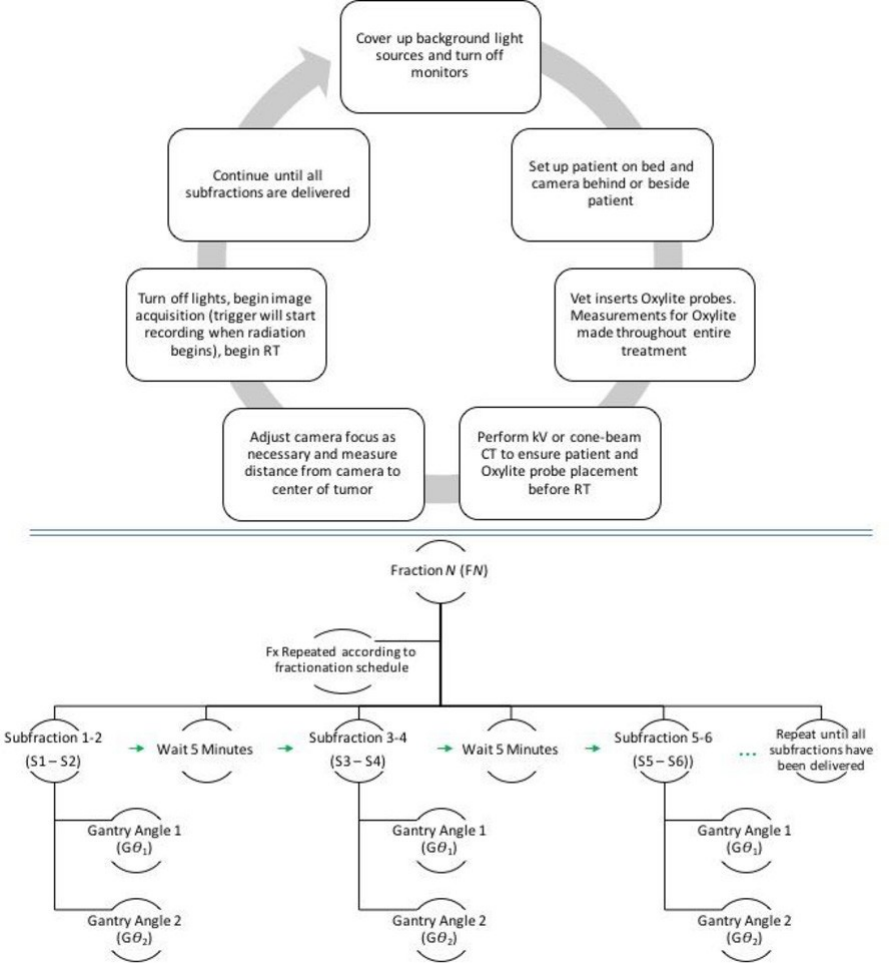
RT and Cherenkov-imaging workflow. One of our aims was to determine if integration of this camera system with the RT workflow is achievable. We found the workflow to be simple. Once the camera was setup, the imaging required just two extra steps: (1) turning off the room lights for the duration of the RT fraction; and (2) pressing the acquisition button in the software. For this study, we simply observed biology that occurred during a standard radiation dose fraction; however, to maximize the amount of data that could be acquired per dog; the individual dose fraction was broken into several equally-sized subfractions that were each given at a standard dose rate (600 monitor units per minute) but temporally separated by 5 minutes.

For radiation delivery between each pair of subfractions, five minutes elapsed to verify that the setup had not changed and to change any beam wedges. See **Fig 2**for the full acquisition and radiation delivery steps.

### Image analysis

**Fig 3:**
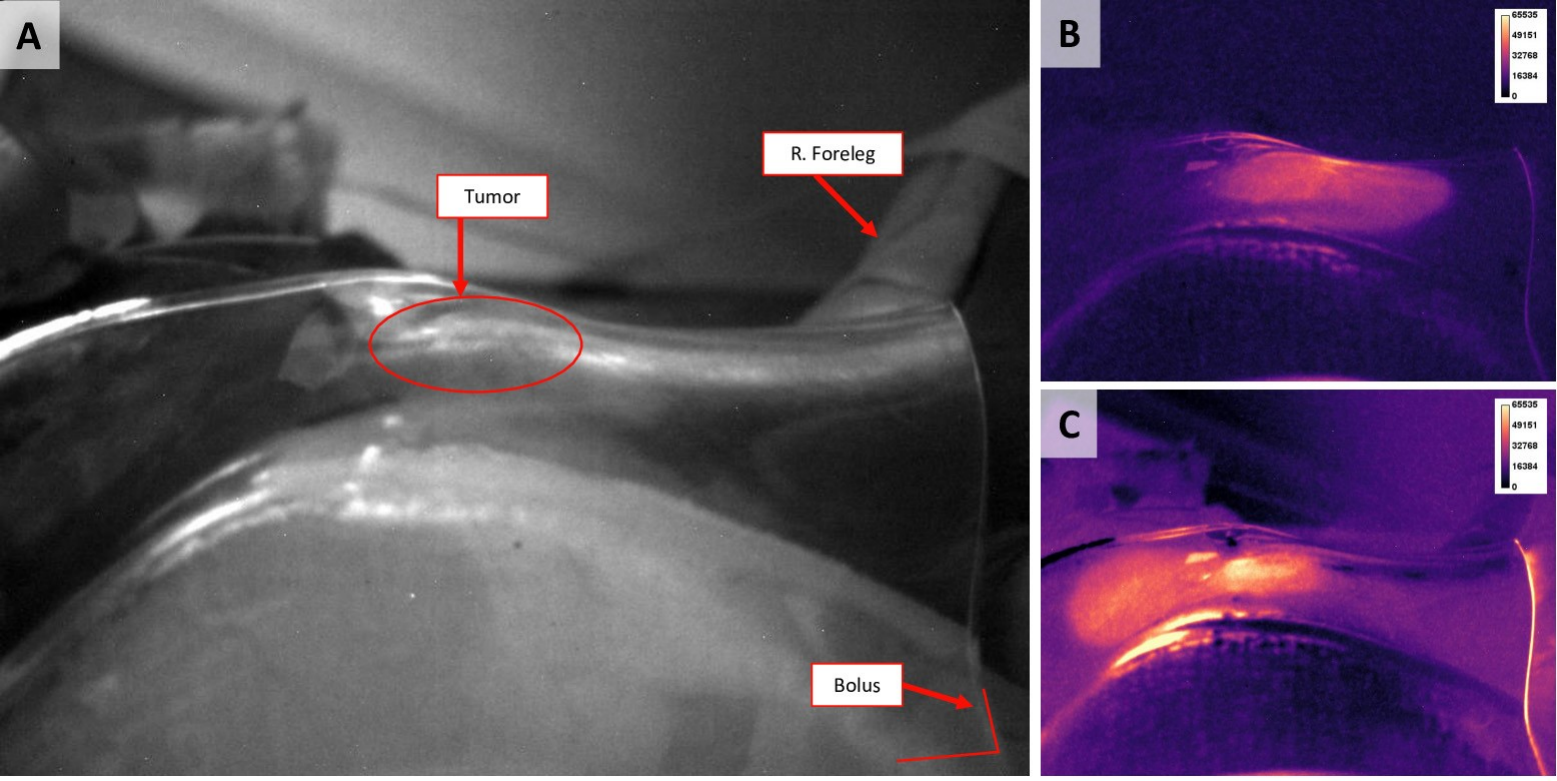
Anatomical, Cherenkov and CT images of the first canine subject. **A)** Representative low-light anatomical (non-Cherenkov) image of canine subject 1 (see **Table 1**), acquired during the second subfraction. The tumor, foreleg and the caudal ventral edge of bolus are labeled in red. **B)** Here, the same dog is depicted but with the Cherenkov images. The gantry was angled at 40˚. **C)** The Cherenkov image corresponding to the anatomical image in A). The gantry angle is at 290˚, and there is a clear dependence of Cherenkov signal on the gantry angle.

Once the data acquisition was completed, our analysis steps were as follows: 1) We analyzed the Cherenkov images by processing them through a Matlab (MathWorks, v. 2018b) program that extracts the images and subtracts the dark background. For every Cherenkov or anatomical image frame (see **Fig 3**), there was a dark background image subtracted from it to account for negligible electronic noise for each channel. In addition to the dark background, the anatomical (i.e.: background) image was acquired for every Cherenkov frame and subtracted. The noise this background image included was predominantly ambient room light from equipment. 2) The Cherenkov frames taken over the course of a ~30s subfraction were analyzed for variability. We found that despite some noise, the tumor intensity was essentially constant throughout each subfraction for each subject. During this process, we measured regions-of-interest around the whole tumor, edge of the tumor, center of the tumor and normal tissue for each subfraction. 3) These Cherenkov frames were averaged over each subfraction and normalized to 1/R^2^ where R is the distance from the center of the tumor to the camera. To compare relative changes in Cherenkov intensity, the signal was normalized to the normal tissue’s first subfraction. 4) The Oxylite data was exported and analyzed similarly with the average tumor or normal tissue pO_2_ calculated across each subfraction. 5) For the Cherenkov data, the inter-subfraction difference was calculated from the appropriate gantry angle. For instance, fraction 3, subfraction 3, gantry angle 1 was subtracted from fraction 3, subfraction 5, gantry angle 1 where subfractions 1, 3, and 5 were at gantry angle 1 and subfractions 2, 4, and 6 were at gantry angle 2. 6) The magnitude of percent change was analyzed for the Oxylite and Cherenkov data. The data from all fractions and canines was combined. The coefficient of variation was calculated by dividing the mean pixel value across the whole tumor by the standard deviation.

Because the water-equivalent bolus, while mostly transparent to Cherenkov emissions, could still absorb some Cherenkov light, all ROIs were drawn within the bolus, and every animal employed the bolus for dose-buildup. The specific fraction of bolus-absorbed Cherenkov emissions was not explicitly quantified–instead it was accounted for by being present in every dataset.

We obtained whole-tumor and Oxylite values for five dogs and whole-tumor, center tumor, tumor edge and Oxylite values for three dogs that completed their entire RT course and whose tumors we could clearly delineate between normal tissue, tumor edge, tumor center and whole tumor. One dog was excluded because its acquisition settings were different than the remaining animals. This was the first animal that we were presented with, so the data was not obtained in a uniform manner compared to the other animals. In addition, a software update was performed after this first dog and before the remaining five dogs. The other five dogs all had data acquired under the same settings.

### Statistical analysis

All statistics were performed in Prism (Graphpad Software Inc. v. 7.0). A one-way ANOVA followed by Tukey’s Post Hoc test was used to determine statistical difference (p<0.05) between tumor areas and normal tissue. A paired, two-tailed T-test was performed to analyze differences between pO_2_ values in the normal tissue and tumor tissue via the Oxylite system with p<0.05 indicating a significant difference.

## Results

### Cherenkov emission images

Anatomical and Cherenkov images were collected for each subfraction and canine subject. **Fig 3**shows an example of the images collected, with **Fig 3A** showing the low-light anatomical image. This background image was taken for each frame during RT, and the average of these frames is shown here with the tumor, right foreleg and bolus clearly presented. **Fig 3B** is the average Cherenkov image across all frames for one subfraction at a gantry angle of 40°, and **Fig 3C** is the same fraction but at a different gantry angle of 290°. Comparing **Fig 3B and 3C**, there is a significant difference in the pattern of Cherenkov emissions based on where the photon beam enters the tumor.

### Quantitative analysis of Cherenkov and Oxylite data

**Fig 4:**
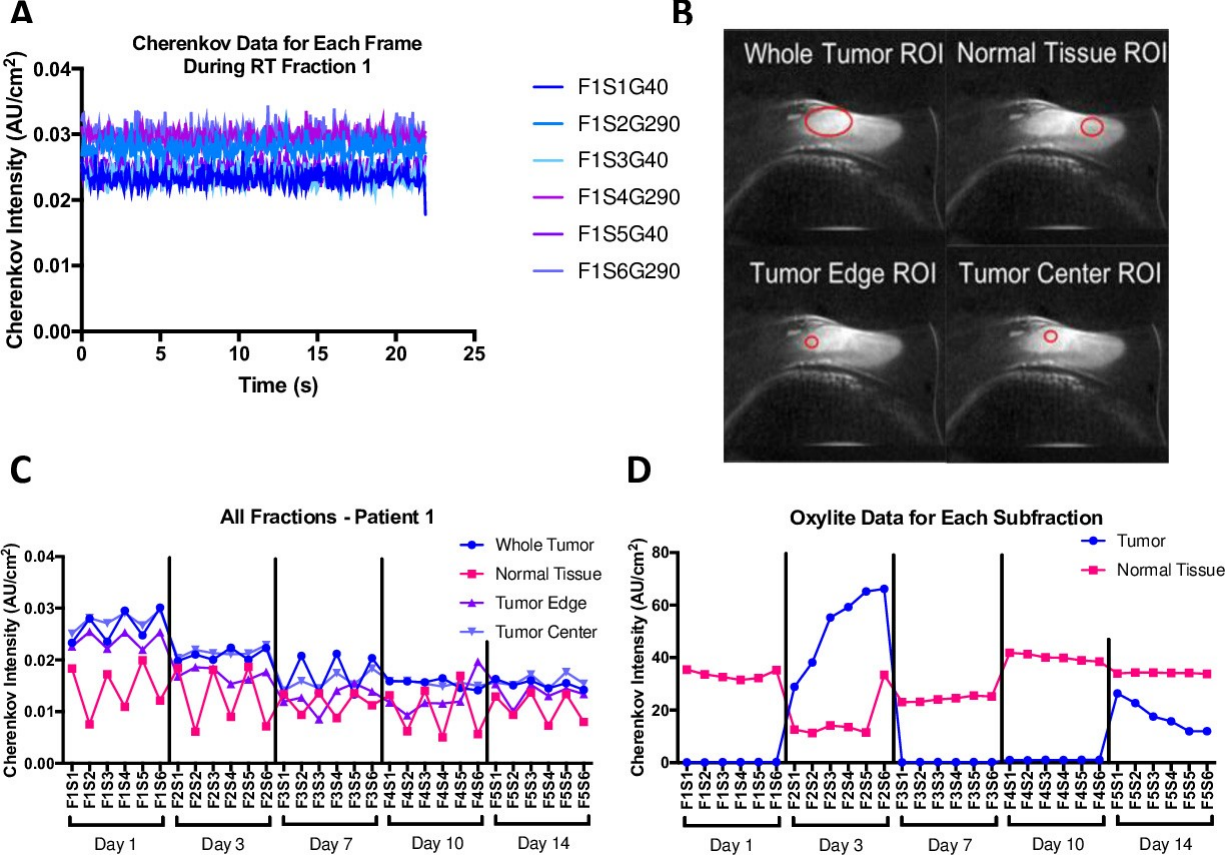
Example data from canine subject #1. **A)** For the whole tumor ROI, the Cherenkov intensity was measured for each frame, normalized to 1/R^2^ and plotted against time. The legend denotes fraction 1 (F1), subfractions 1–6 (S1-S6), and gantry angles 40° and 290° (G40 and G290). Over time, each of the frames is consistent, suggesting that average Cherenkov information for each subfraction is a good representative of the data. **B)** For canine 1, fraction 1, subfraction 1, gantry angle 40°, the ROIs are drawn in red around the whole tumor, normal tissue, tumor edge and tumor center. **C)** For the same dog, the averaged value for each subfraction is recorded with the standard error of the mean as the error bars. The vertical black bars denote different fractions on different days. **D)** Similarly, the Oxylite data is shown for both the tumor and normal tissue over the course of the entire treatment. Each data point represents the mean Oxylite value for a subfraction.

**Fig 4**shows an example of the Cherenkov data collected for each canine subject. **Fig 4A** shows the Cherenkov intensity of the whole tumor over each frame for Fraction 1 where each data point has been normalized to 1/R^2^ where R is the camera-tumor distance. In our first data set (case #6 in **Table 1**), we saw periodic peaks in the data that indicated a blinking light source was contaminating the Cherenkov data. Future acquisitions used a frame-by-frame background subtraction method to ensure overcome this issue. **Fig 4B** shows the next step in analyzing the data–drawing ROIs around the whole tumor, normal tissue, tumor center and tumor edge. The quantification of these ROIs is shown for canine subject 1 across all fractions and subfractions in **Fig 4C**. Note the oscillations in the data dependent on the gantry angles. The Oxylite data is shown in **Fig 4D** for the same canine across the entire RT plan.

**Fig 5:**
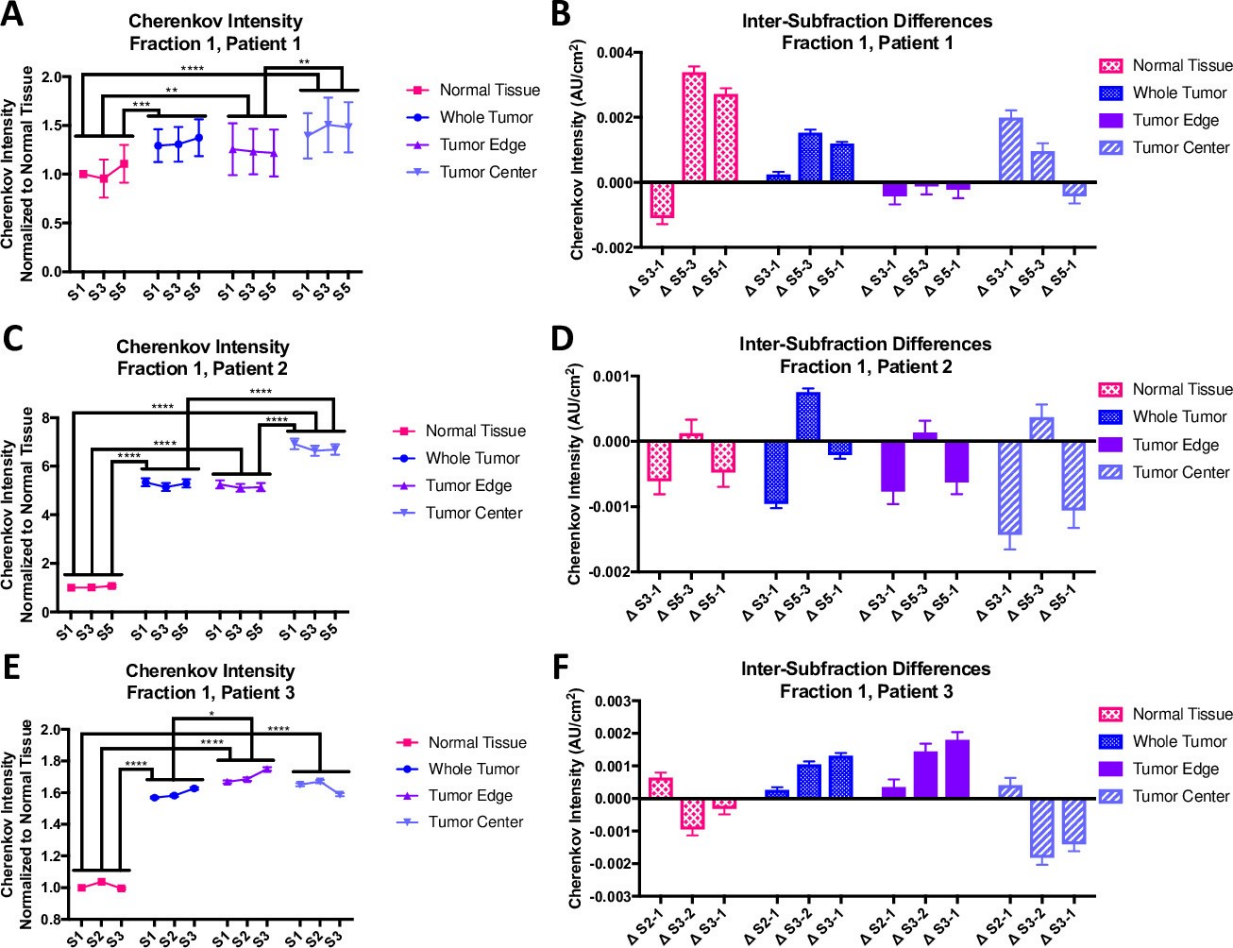
First fraction data for three canine subjects. The Cherenkov intensity data (A, C, E) has been normalized to the first normal tissue subfraction. The intensity for the whole tumor, normal tissue, tumor edge and tumor center is shown, all for the same gantry angle. Using a one-way ANOVA that compared all groups followed by Tukey’s multiple comparisons test, normal tissue is consistently different than the remainder of the tumor areas, as is the tumor edge (* p<0.05, ** p<0.01, *** p<0.001, **** p<0.0001). The intensity difference between the adjacent subfractions as well as the difference between the first and last subfractions are shown (B, D, F). No significant difference between ΔS_X2_-S_X1_ was found nor are there any trends in the data. **A)** Normalized Cherenkov intensity for canine 1, fraction 1 **B)** Inter-subfraction difference for canine subject 1, fraction 1 **C)** Normalized Cherenkov intensity for canine subject 2, fraction 1 **D)** Inter-subfraction difference for canine subject 2, fraction 1 **E)** Normalized Cherenkov intensity for canine subject 3, fraction 1 **F)** Inter-subfraction difference for canine subject 3, fraction 1.

**Fig 5**summarizes data from Fraction 1 for three canine subjects. The left column (**Fig 5A, 5C and 5E**) plots the average Cherenkov intensity normalized to subfraction 1 (S1) of the normal tissue with the standard deviation. A one-way ANOVA followed by Tukey’s post-hoc test was used to analyze the differences between tissue types. For all animals, the normal tissue was significantly different from the tumor (* p<0.05, ** p<0.01, *** p<0.001, **** p<0.0001). There is some consistency with the center of the tumor being significantly different than the tumor edge; likewise, the whole tumor is different from the tumor center (**Fig 5C**) and tumor edge (**Fig 5E**). The right column of graphs (**Fig 5B, 5D and 5F**) shows the difference in Cherenkov intensity from the first subfraction. There is no clear trend in the difference increasing or decreasing in a single fraction for a single tissue.

**Fig 6:**
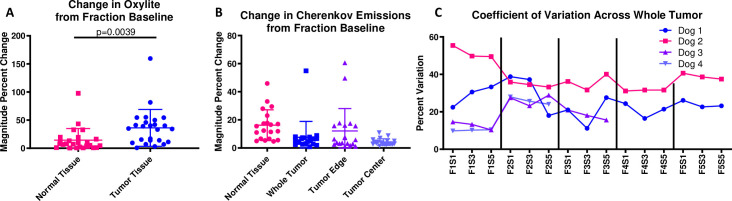
Summary of percent change from fraction baseline for included canine subjects. For each fraction FN, the magnitude of the percent change was calculated for three canine subjects and combined into a single graph. **A)** The Oxylite data for normal tissue and tumor tissue are significantly different according to a two-tailed, paired T-test with p = 0.0039. Though the Oxylite data is only a point measurement, it is clear that tissue oxygen levels are changing more in the tumor than in the normal tissue. **B)** The Cherenkov data shows that the absolute percent change for the tumor edge is the largest. The normal tissue also changes from baseline while the whole tumor and tumor center does not change as much, relative to the other tissue areas. **C)** The coefficient of variation was calculated for each whole tumor over the course of the entire radiation plan for a single gantry angle (standard deviation / mean). Not only is there high pixel-pixel variation, but that variation changes for a single fraction.

**Fig 6A and 6B** summarizes the Oxylite and Cherenkov data as the magnitude of percent change from the baseline (subfraction 1) for a fraction. A two-tailed, paired T-test reports a significant difference between the magnitude in percent change in normal tissue Oxylite values compared to tumor values (p = 0.0039) (**Fig 6A**). **Fig 6A/6B** include data from all three canine subjects. **Fig 6B** showed that the normal tissue and tumor edge change the most compared to baseline. **Fig 6C** describes the coefficient of variation for all analyzed canines. The coefficient of variation is high for a static image; the radiation dose rate is constant, so pixel-by-pixel differences might be accounted for by the different Cherenkov emission and absorption coefficients in different types of tissue.

## Discussion

### Integration of non-invasive, functional imaging into the clinical workflow

The DoseOptics camera was easily integrated into the clinical workflow, but not without several challenges. Light contamination by monitors and other sources in the room needed to be covered prior to image acquisition. The lights needed to be off during dose-delivery, which was challenging as the anesthesia monitoring in the LINAC suite was through closed circuit camera. This is generally less of a concern for adult, human cancer patients undergoing RT, or if the monitoring equipment were located outside of the LINAC vault, in the control room. While the RT schema we used included sub-fractionated fractions, we expect that standard fractionation schemes would provide sufficient data for analyzing the Cherenkov emissions spectra without resorting to comparisons made between small subfractions.

This paper describes the simplest possible scenario of unmodulated EBRT. The next step in integration is determining if the camera can be used in the setting of uneven radiation fluence (i.e., IMRT). If the camera can be placed on the table, the gantry would be free to move to any angle, and IMRT would be feasible. For the cases where the tumor cannot be visualized from that angle, IMRT might be more challenging. In a human clinic, it would be challenging to place a camera on the table (there would be very little room). One alternative is to mount the camera onto an omnidirectional arm from the ceiling. The camera could then be placed at any angle, either about the table or from behind the gantry (above the LINAC). This method would enable delivery of a complex RT plan.

The DoseOptics camera was originally developed to monitor the dose during RT. In the setup described previously with a ceiling-mounted camera, this additional application would useful for confirming how much dose is delivered, though using this camera as an optical dosimeter is fraught with its own challenges owing to gantry angle, beam details and tumor tissue [21–23]. The other application of monitoring functional tumor changes, as investigated by this study, would provide much-needed information about the changes a tumor undergoes during RT. As a non-invasive, relatively inexpensive, and easily-integrated system, there are few downsides to including additional data acquisition during radiation delivery.

### From Cherenkov emission images to functional imaging

The results of the Cherenkov emission images in different areas of the tumor and normal tissue suggest that there is a biological cause for the changes in signal. The whole tumor, edge of the tumor and center of the tumor were all different, sometimes significantly different, despite a homogenous radiation beam being delivered within the PTV. There is evidence of Cherenkov intensity depending on the angle of the beam, the angle of the camera and the tissue surface curvature [24, 25]. We did not quantitatively explore angular dependency; however, the comparisons between different tumor regions were done so in the same fraction, under the same gantry angle, and where the camera remained in the same position. This minimized angular dependency of Cherenkov intensity due to gantry angle or camera angle relative to the tumor; the gantry and camera angle account for a significant portion of the angular dependency in Cherenkov intensity. There is additional evidence that tissue shape influences Cherenkov intensity [21]. The tumor curvature in our studies was not as steep as those described in a study by Zhang, et al, [21] where they studied the effects of Cherenkov intensity in a cylindrical phantom. In addition, our tumors were not subject to the lack of depth at the edge of the cylinder. As such, in this pilot study, the effects of tissue curvature were largely ignored; however, this should be investigated extensively in future studies.

An additional contributor to Cherenkov signal is fluorescence. Cherenkov-excited fluorescence has been measured [26, 27]; however, we expect the fluorescent signal to be small compared to the Cherenkov intensity. In order to measure fluorescence, even signal emitting from bright probes, efficient, small-bandpass emission filters are necessary to filter out the excitation light. As such, while fluorescence contributes to our Cherenkov signal, the ability to measure this small portion of the signal would not be possible with the DoseOptics camera and without specialized equipment [27]. For this pilot study, fluorescent contribution was ignored.

In addition to the physical descriptors of the tumor such as size, shape, and melanin content (all contenders as sources of variation across the tumor) and fluorescence, internal biology also could play a role in heterogenous Cherenkov emission signal across a tumor. Solid tumor modeling shows that the periphery of a tumor tends to have dense vasculature due to continual angiogenesis in invasive regions [28, 29]. This would result in a different spectral weight of oxygenated hemoglobin in our images. However, density of vasculature is not the only cause for the differences we report. The other dominant absorber in solid tumors at this frequency range is water. According to a recent study where Raman spectroscopy was used to delineate the border between malignant and healthy tissue in oral cavity squamous cell carcinoma, water content was the primary factor in describing the tumor margins with a 99% sensitivity and 92% specificity [30, 31]. A clinical study compared hemoglobin, water and lipid content in breast tumors receiving neoadjuvant chemotherapy [32]. The results suggest that diffuse optical spectroscopy, when applied to quantifying these tissue components, was a good predicter of response. The spectral components of tumors provides powerful information about the biology and functional status. It is possible to isolate the spectral components of oxygenated versus deoxygenated hemoglobin from Cherenkov emissions [17, 18]. Notably, the hemoglobin and water absorption spectra are well-separated, which would make either option feasible for quantifying either component of tumor tissue via Cherenkov emission imaging.

The success of optical methods for obtaining biological and physiological information about tumors is based upon the ability to distinguish transmission, absorption and scattering components. To do so, there is a level of signal-to-noise that needs to be achieved so that filtering the signal to only obtain hemoglobin saturation data, for instance, will not result in a noisy dataset. Our average signal-to-noise ratio (SNR) is 14dB. We expect that any future spectral acquisitions via optical filters will result in a high enough signal to analyze. Moreover, the coefficient of variation across the pixels of a given tumor is also indicative of a good signal because the intensity differences are reflecting the different absorption components in the tumor. We would expect that a homogenous or noisy sample would have relatively low variation; however, **Fig 6C** reveals substantial variations from pixel-to-pixel across a tumor, likely stemming from biological inhomogeneities across the tumor. Real-time monitoring of biological changes during radiation therapy via Cherenkov imaging has implication for predicting therapeutic efficacy and improving therapeutic response.

Cherenkov imaging adds another factor to consider: dose. Our results of consistent, highly significant differences between tumor and normal tissue could be a result of the differences in tissue components (like water). However, while we quantified regions of normal tissue that received some dose, the normal tissue was not included in the GTV, so the decrease in normal tissue intensity compared to tumor tissue intensity is reflected in the lower dose delivered to the edge of the PTV. If, in future studies, we choose to quantify the biology of the tumor and normal tissues, it would be important to account for dose variations based on the tissue. Either way, the camera is sensitive enough to detect intensity changes as the dose decreases near the edge of the primary beam.

## Conclusion

Despite some setup challenges, the DoseOptics system was smoothly integrated into the clinical workflow. Six different canine subjects presented with unique radiotherapy treatments and tumor sizes/areas, and a workable camera setup was achieved for each (3/6 datasets were used for extensive analysis and 5/6 datasets were used for summary analyses). The results of this pilot study show that there are significant intra-subfraction differences in the Cherenkov intensities for normal tissue, whole-tumor tissue, tissue at the tumor edge and tissue at the tumor center. There is a complex relationship between Cherenkov intensity and blood-oxygen saturation that could be dependent on many physiological variables, like the heterogeneous distribution of oxygen across a tumor. By extending this study to a larger cohort and obtaining additional spectral data, this technology could potentially be extended to non-invasively reporting real-time changes in the tissue, the water content, and tumor pO_2_ during radiotherapy.

## Supporting information

S1 File (PZFX)
